# Characterization of Variant RNAs Encapsidated during Bromovirus Infection by High-Throughput Sequencing

**DOI:** 10.3390/pathogens13010096

**Published:** 2024-01-22

**Authors:** Sarah Dexheimer, Nipin Shrestha, Bandana Sharma Chapagain, Jozef J. Bujarski, Yanbin Yin

**Affiliations:** 1 Department of Biological Sciences, Plant Molecular and Bioinformatics Center, Northern Illinois University, DeKalb, IL 60115, USA; sarahdex13@gmail.com (S.D.); nipin.shrestha@gmail.com (N.S.); bandanaschapagain@gmail.com (B.S.C.); 2 Nebraska Food for Health Center, Department of Food Science and Technology, University of Nebraska—Lincoln, Lincoln, NE 68588, USA

**Keywords:** RNA viruses, RNA-seq, mutation profiling, RNA recombination, substitutions, deletions, SNPs, Bowtie, ViReMa

## Abstract

Previously, we described the RNA recombinants accumulating in tissues infected with the bromoviruses BMV (Brome mosaic virus) and CCMV (Cowpea chlorotic mottle virus). In this work, we characterize the recombinants encapsidated inside the purified virion particles of BMV and CCMV. By using a tool called the Viral Recombination Mapper (ViReMa) that detects recombination junctions, we analyzed a high number of high-throughput sequencing (HTS) short RNA sequence reads. Over 28% of BMV or CCMV RNA reads did not perfectly map to the viral genomes. ViReMa identified 1.40% and 1.83% of these unmapped reads as the RNA recombinants, respectively, in BMV and CCMV. Intra-segmental crosses were more frequent than the inter-segmental ones. Most intra-segmental junctions carried short insertions/deletions (indels) and caused frameshift mutations. The mutation hotspots clustered mainly within the open reading frames. Substitutions of various lengths were also identified, whereas a small fraction of crosses occurred between viral and their host RNAs. Our data reveal that the virions can package detectable amounts of multivariate recombinant RNAs, contributing to the flexible nature of the viral genomes.

## 1. Introduction

Plus-sense RNA viruses rapidly adapt to new environments, and the processes of RNA recombination diversify viral populations [1,2]. Homologous and non-homologous RNA recombination can lead to the accumulation of beneficial mutations but can also eliminate the harmful ones. These recombinations can reshuffle larger portions of the viral genome, generating defective RNAs, or cause crossovers among different viral or viral and host RNAs [3,4]. RNA recombinants have been described in various animal viruses [5,6,7,8,9,10], plant viruses [11,12], bacteriophages [13], negative-sense RNA viruses [14], and retroviruses [15]. Defective (D) or defective interfering (DI) RNAs have been identified for both animal and plant RNA viruses, either in natural infections or in cell culture.

Bromoviridae are tripartite plus-stranded RNA viruses of plants. The Brome mosaic virus (BMV) is one of the best-characterized bromoviruses. Its virions separately encapsidate RNA1, RNA2, or RNA3, plus subgenomic [sg] RNA4 components [16]. RNA recombination has been studied extensively for BMV. Both homologous and nonhomologous crossovers were detected among RNA segments or within the same RNA segment [4]. Mapping the cross sites revealed recombination hot spots near the secondary structures [17], while mutations in 1a and 2a proteins changed the crossover hotspots [18,19]. RNA recombination was also demonstrated in the Cowpea chlorotic mottle virus (CCMV) using transgenically-expressed viral RNA fragments [20]. These and other observations strongly suggested replicase-, but also the coat protein (CP)-, mediated template switching processes [16,21,22,23].

The various kinds of BMV RNA recombinants described to date were analyzed in total RNA extracts from the infected tissue. We previously described that BMV and broad bean mottle virus (BBMV) virions could package the host cellular RNA sequences [24,25]. To find out if virions could package the recombinant variants, in this work we analyzed BMV or CCMV RNAs extracted from extensively purified viral preparations. By using the techniques of high-throughput sequencing (HTS) and a recently developed algorithm named Viral Recombination Mapper (ViReMa), described in [26], we characterized groups of encapsidated recombinant RNAs. These included intra- and inter-segmental crossings, virus-to-host recombinants, as well as larger insertions and substitutions. Overall, our results reveal populations of RNA variants (RNA cloud) that can be carried in viral particles, in addition to their canonical genomic RNA components.

## 2. Materials and Methods

### 2.1. Virus Propagation, Purification, and RNA Extraction

BMV (Russian strain) was propagated in barley seedlings and purified by ultracentrifugation in a sucrose gradient, essentially as described in [27]. Similar protocols were utilized for purification of CCMV (Bawden strain) that was grown in cowpea seedlings (cv. California blackeye #5). After collecting the distinct opalescent virus bands from sucrose gradients, the virions were pelleted down and the glassy pellet re-suspended in the virus buffer (VB; 0.05 M NaOAc, 0.008 M Mg(OAc)_2_, pH 4.5), by soaking overnight. Then, the virus preparations were treated with RNase and DNase to eliminate co-purifying host nucleic acids, and the nucleases were removed by centrifugal concentration in Amicon Ultra 4–5 mL concentrators (UFC810024), as described in [24]. The viruses were finally washed and re-suspended in storage buffer (10× diluted VB). The purified virions were lysed and the encapsidated viral RNA extracted and purified, as described in [24]. The final RNA preparations were dissolved in RNase-free water, and the integrity of the RNA was confirmed by electrophoresis in a denatured (formamide/formaldehyde) agarose gel (not shown).

### 2.2. High-Throughput Sequencing (HTS)

To prepare the RNA sequencing library, viral RNAs were treated with the PrepX RNA-Seq Library Kit by Wafergen Biosystems (Fremont, CA, USA). Briefly, the protocol involved limited digestion with the RNaseIII enzyme, followed by the cDNA synthesis with the superscript III reverse transcriptase, PCR amplification with LongAmp Taq polymerase and primer mix, and purification of the library. For details see at http://www.wafergen.com (accessed on 15 January 2024) and Takara.com (accessed on 15 January 2024). This non-stranded RNA-Seq library preparation allowed us to address global reads at both direct and complementary cDNA levels. The nucleotide sequences were then determined on an Illumina HiSeq2000 instrument (Illumina, San Diego, CA, USA), at the University of Illinois at Chicago, Center for Genomic Research (http://tinyurl.com/dnas-ilab) (accessed on 15 January 2024).

### 2.3. Sequence Analysis with Bioinformatics Tools

The HTS data were then analyzed with bioinformatics tools. First, to avoid incorrect assemblies, low-quality score reads (*p* < 0.05) as well as short ones (<50 nts) were eliminated by using DynamicTrim and LengthSort v. 2.5 in Solexa QA software [28]. Then the cleaned reads were mapped to both viral RNA genomes published in GenBank, shown in Table 1, and the plant host mRNAs and rRNAs: (i) BMV host *Hordeum vulgare* (barley), from JGI (https://phytozome.jgi.doe.gov/pz/portal.html#!infoalias=Org_Hvulgare_er) (accessed on 15 January 2024); (ii) CCMV host *Vigna unguiculata* (cowpea), from JGI (https://phytozome.jgi.doe.gov/pz/portal.html#!info?alias=Org_Vunguiculata_er) (accessed on 15 January 2024). The cleaned BMV and CCMV reads were deposited into GenBank (PRJNA565451).

**Figure 1:**
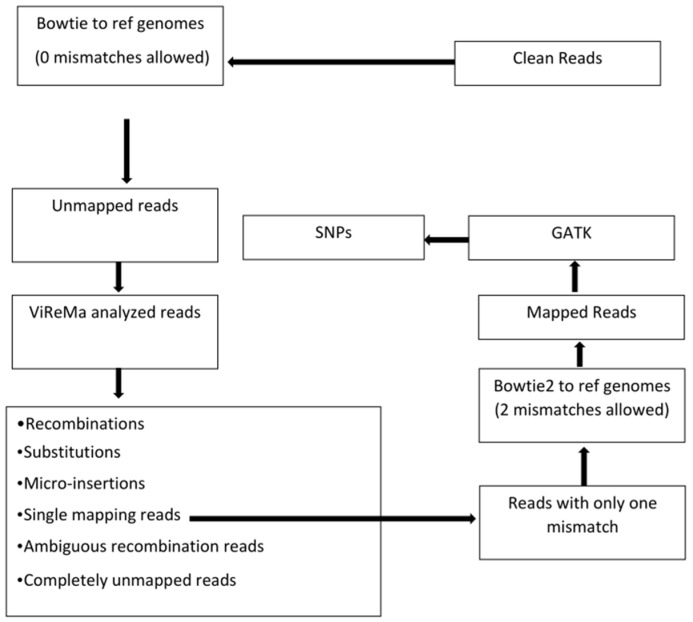
Flow chart of bioinformatics data analyses. The reference genomes for Bowtie mapping and ViReMa analysis include not only the viral genomes but also plant hosts’ transcriptomes (mRNAs and rRNAs). Bowtie version 1 was used before ViReMa analysis because Bowtie1 allows a perfect match without mismatches, and we aimed to collect unmapped reads with all kinds of variations for ViReMa analysis. Only reads indicated to have one mismatch from single alignment reads were fed into Bowtie2, which allows mismatches for SNP identification. The different categories of read outputs from ViReMa are explained in Section 3.

As outlined in Figure 1, the trimmed reads were mapped to the reference sequences (viral genomes and the host transcriptomes) using Bowtie version 1 [29] with the following command: *bowtie –v 0 –S reference_index fastq_file –un unmapped_read_output_file mapped_read_output_file*. Mismatches allowed were set to zero. The results from Bowtie mapping are shown in Table 2.

**Figure 2:**
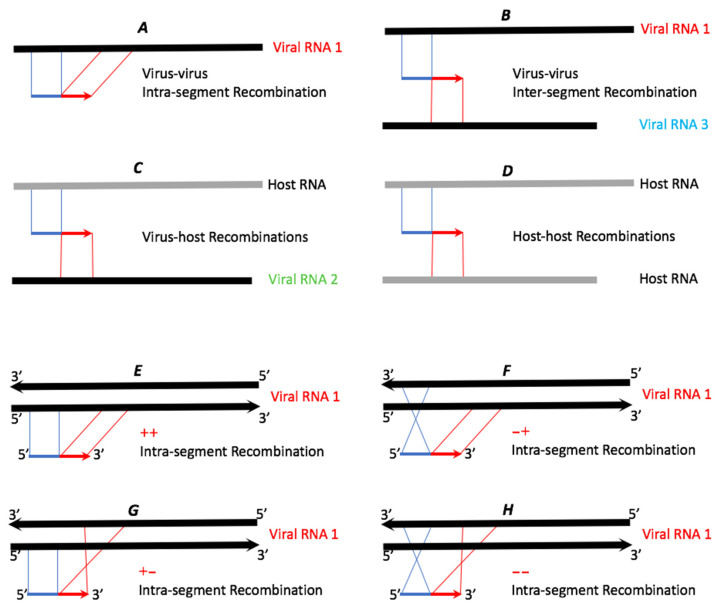
Schematic examples of potential types of recombinant reads. The thicker lines symbolize reference RNAs (black for viral RNAs and grey for host RNAs). The thinner blue and red lines represent recombinant reads, with blue (5′) and red (3′) representing two recombined portions of the reference RNAs. Please note that since the non-stranded sequencing library was prepared, cross types E and H cannot be assigned to the replicating + or − viral RNA strands.

The Bowtie unmapped reads were processed by ViReMa [26]. Instead of mapping the full-length reads, the ViReMa algorithm attempts to split the reads and iteratively maps the 5′ and 3′ segments of the reads to the references using Bowtie. Hence, ViReMa identifies reads that are derived from (i) recombination crosses as shown in Figure 2; (ii) small insertions; (iii) small deletions (≤5 nts); (iv) multi-base substitutions (alignment with pads—a short stretch of nucleotides (<25 nts) that did not align to reference), and (v) completely unmapped reads. ViReMa was run with default parameters by using the viral genome and the host transcriptome (mRNAs + rRNAs) as the reference index. The statistics of ViReMa result can be found in Table 3.

In order to identify SNPs, the output file from ViReMa was parsed to extract reads with only one mismatch (1×) and no other unmapped regions. These reads were then extracted and subjected to another round of Bowtie mapping with two allowed mismatches. Bowtie-mapped reads were then analyzed using the Genome Analysis Toolkit [30] in order to identify single nucleotide polymorphisms (SNPs).

Unix Bash and Perl scripts were developed to parse the output files from ViReMa to generate data for graphs. For all figures, only the events with greater than 10 supporting reads were used. We also tried plotting events with greater than 5 supporting reads, and the conclusions did not change. R version 3.2.5, specifically the ‘ggplot2’ package (https://ggplot2.tidyverse.org/) (accessed on 15 January 2024), was used to make figures.

**Figure 3:**
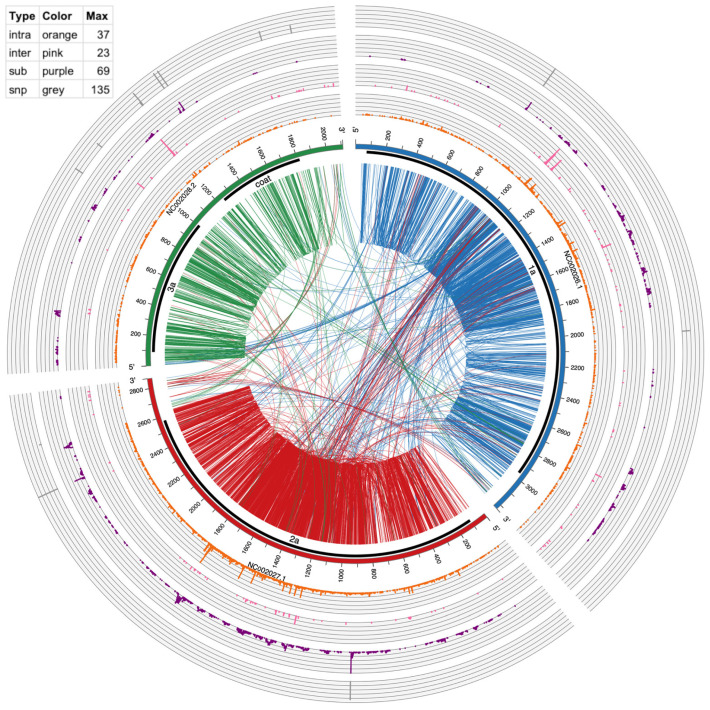
Circos plot of mutation events in BMV RNAs. From outside to inside: SNPs (gray bars), multi-base substitution events (≥10 supporting reads, purple histogram), inter-genome recombination (≥10 supporting reads, pink histogram), intra-genome recombination (≥10 supporting reads, orange histogram), genome ideogram (with ticks marking the nucleotide position), protein coding region shown as thick black lines, and encoded protein product labels, lines connecting two positions within an RNA (intra-genome crosses) and between two RNAs (inter-genome crosses). These recombination lines are directional, being color-coded according to which RNA the line starts from. For example, lines starting from NC_002026 are coded in dark blue, representing that the recombination has its 5′ end in this RNA. The table in the top left shows the height and spacing of the four histograms, representing the number of events found in each position. Higher means more events or hot mutation spots.

**Figure 4:**
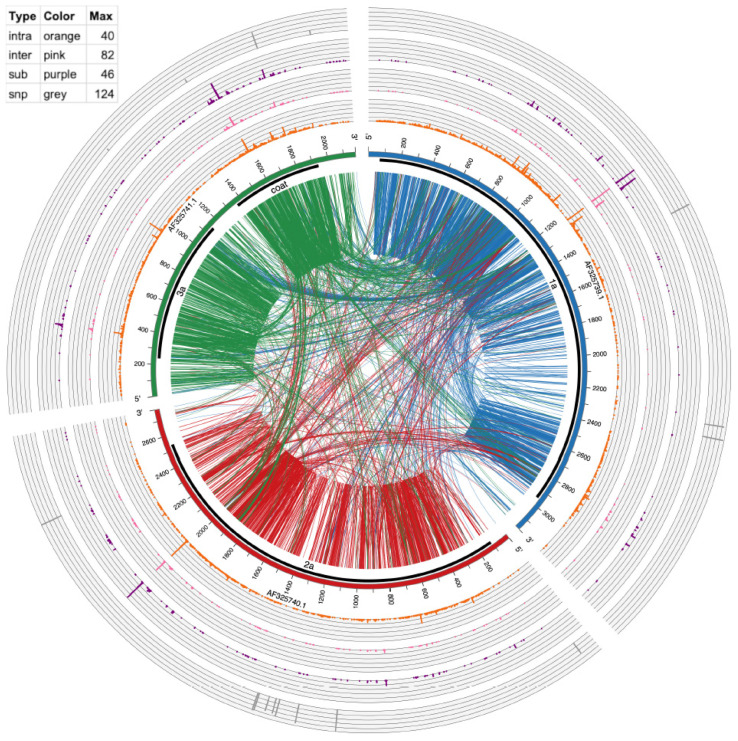
Circos plot of mutation events in CCMV RNAs. From outside to inside: SNPs (gray bars), multi-base substitution events (≥10 supporting reads, purple histogram), inter-genome recombination (≥10 supporting reads, pink histogram), intra-genome recombination (≥10 supporting reads, orange histogram), genome ideogram (with ticks marking the nucleotide position), protein coding region shown as thick black lines and encoded protein product labels, lines connecting two positions within an RNA (intra-genome crosses) and between two RNAs (inter-genome crosses). These recombination lines are directional, being color-coded according to which RNA the line starts from. For example, lines starting from AF325739 are coded in dark blue, representing that the recombination has its 5′ end in this RNA. The table in the top left shows the height and spacing of the four histograms, representing the number of events found in each position. Higher means more events or hot mutation spots.

Circos version 0.69–5 [31] was used to make Figure 3 and Figure 4. The links in the inside of the plot visualize actual recombination events. The circular graphs around the outside of the plot represent frequency of substitutions, inter-recombination, and intra-recombination crosses. Intra-recombination events were defined as those occurring within the same viral segment (e.g., RNA1); inter-recombination events were defined as events between two different viral segments (e.g., RNA1 and RNA2).

## 3. Results

### 3.1. High Numbers of HTS Reads Do Not Map to the Canonical Viral Genomes

Two bromoviruses, BMV and CCMV, were propagated in barley and cowpea plants, respectively, and purified, and the encapsidated viral RNAs were extracted, as described in Section 2. The RNAs were subjected to high throughput sequencing, generating over 100 million 76 nt single-end reads as shown in Table 2 (NCBI BioProject ID: PRJNA565451). Considering the total RNA genome size of 8210 nts for BMV and 8122 nts for CCMV, the sequence depth was over 10^6^, enough to detect very rare mutations (rate 10^−6^).

The reads were mapped to the published references by applying a developed analysis pipeline (Figure 1). The use of the Bowtie program revealed nearly 70% reads mapping to the viral RNAs, but at different proportions per RNA segment, reaching respectively 22.63%, 40.17%, and 8.28% for BMV, and 34.69%, 16.08%, and 16.78% for CCMV RNAs as shown in the Table 2. The different ratios of encapsidated viral RNAs could be due to different molecular requirements, e.g., during RNA replication, translation or encapsidation. Aside from the reads matching the viral RNA sequences, a significant percentage of reads remained to be unmapped for both BMV (28.85%) and CCMV (32.31%), which are the focus of this study.

### 3.2. ViReMa Identified Six Classes of RNA Variants among the Bowtie-Unmapped Reads

The mutations on the viral RNAs generate variant reads that do not map to the reference. Those include point mutations, insertions or deletions, and recombination events. To identify these variants, the Bowtie unmapped reads were further analyzed by ViReMa [26] (see Section 2). As shown in Table 3, ViReMa cataloged the reads into the following six categories

1. Recombination crosses that include reads split into at least two fragments in the reference RNAs (illustrated in Figure 2). The crossovers that occur in the same RNA segment will appear as insertions or deletions (see below). Out of 31,429,207 BMV reads and 35,192,299 CCMV reads, 1.40% and 1.83% were reported as such recombinant reads.
2. Ambiguous recombinants included reads with pads longer than 25 nts at either end, or reads with pads of any size in the middle (12.73% and 9.45% of reads, respectively).
3. Nucleotide substitutions in reads carrying larger than two consecutive nucleotide mismatches. We found that 1.25% and 1.21% reads of BMV and CCMV fell into this group. The single nucleotide mismatches (or SNPs) were not reported by ViReMa (see the additional analysis of SNPs below).
4. Micro-insertions included reads mapped to a single reference after exclusion of a small number (≤4 nts) of nucleotides in the middle, involving 0.01% and 0.23% reads in the two viruses. Reads with longer than 5 nts insertions were reported as ambiguous recombinants by ViReMa.
5. Single mapping reads carried the pads of unmapped regions shorter than 25 nts, as well as reads with single-base mismatches in the middle. Those involved, 80.40% and 82.08% reads for BMV and CCMV, respectively.
6. Unmapped reads. Only 4.21% and 5.20% reads fell into this category.

### 3.3. Inter-Segmental vs. Intra-Segmental Recombinants

Different groups of recombination events illustrated in Figure 2 were parsed from the ViReMa recombination output file of 440,975 and 644,596 reads (Table 3) of BMV and CCMV. Theoretically, the recombinant reads can be derived from intra-segmental or inter-segmental viral crossovers. Additionally, reads can be mapped to RNAs between the host and the virus, or between two RNAs of the host. As summarized in Table 4, the number of recombination events varied, depending on the RNA segment. Among the total events for CCMV (4983) and BMV (4009), the latter supported more events in RNA2, followed by RNA1 and RNA3, whereas in CCMV the order was RNA1, RNA3, and RNA2 (consistent with the Bowtie data of Table 2). In both viruses, there were higher numbers of intra-segmental than the inter-segmental events: 91% (3642 out of 4009) events in BMV, compared to 78% (3888 out of 4983) in CCMV (see also Section 4).

A characteristic of intra-segmental recombinants was that most of them carried short insertions/deletions (indels). Table 5 shows that out of the 3642 intra-segmental crosses in BMV, 1493 led to insertions and the rest to deletions. The 91.8% deletions had a length ≤5 nts. Among 3888 intra-segmental crosses in CCMV, 1365 caused insertions and 2523 caused deletions; 2155 (85.4%) deletions had a length ≤5 nts. The length of indels was either a multitude of 3, 3 plus 1, or 3 plus 2, leading to six types of indels (Table 5). Del1 was the most abundant (42.83% in BMV and 45.65% in CCMV), together with ins2 accounting for over 50% of events and leading to 1->2 frame shift. Overall, most events caused the frameshifts within the coding region. Distribution of length of indels are shown in Supplementary Figure S1.

### 3.4. Mapping of Substitutions to the Reference Genomes

**Figure 5:**
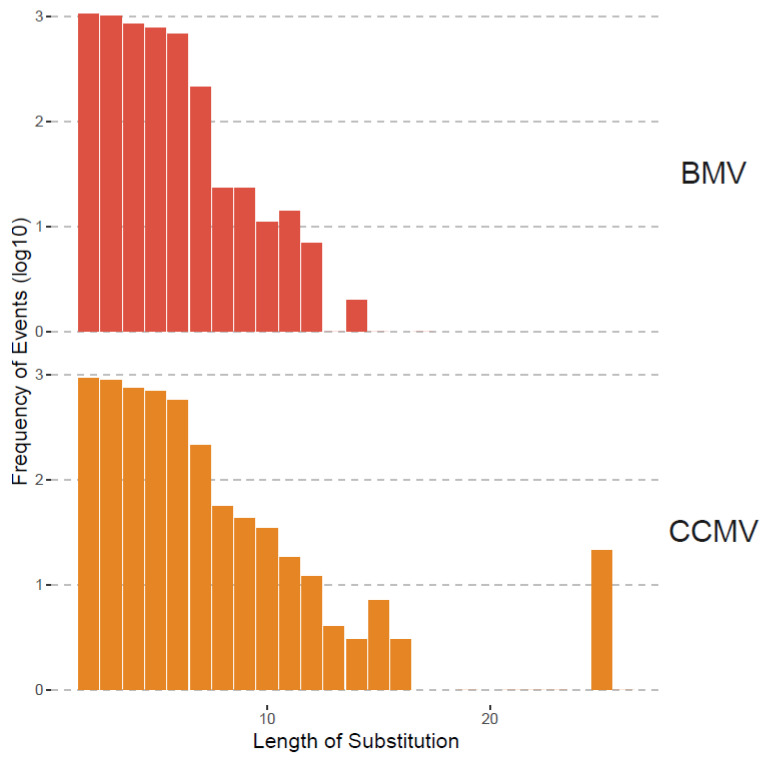
Length distribution of substitutions in BMV and CCMV. The x-axis is the length starting at 2 nts. The y-axis is the frequency (i.e., the number of events having that length of substitution) shown in log10 scale.

Although recombination was the focus of this study, we also analyzed substitutions as shown in Table 3 and Figure 3 and Figure 4. Substitutions were mapped by ViReMa Version 0.6 as mismatches longer than two consecutive nucleotides in the reference genome. Table 3 provides the total numbers of reads derived from such multi-base substitutions, whereas Figure 5 shows the distribution of their length. Apparently, for BMV, the lengths of substitutions are evenly distributed for two to six nts, but gradually diminished to zero at 14 nts and longer. For CCMV, the number of events diminishes at a slower rate to an outlying category at 25 nts.

The largest category of ViReMa-mapped reads were single mapping mismatches, Single nucleotide polymorphisms (SNPs) from these reads were identified by using a separate approach, as described in Section 2. As illustrated in the outermost ring in Figure 3 and Figure 4, after filtering for ≥10 supporting reads, a total of 25 SNPs was found in BMV (RNA1: 4, RNA2: 5, RNA3: 16), and 30 in CCMV (RNA1: 6, RNA2: 18, RNA3: 6). This shows the RNA variants can also arise from the SNPs along with the other recombinations described above.

### 3.5. Visualizing of Recombination Hotspots with Circos Plots

To provide a global mapping of RNA crosses, as shown in Table 4 and Table 6, all inter- and intra-recombination events that were supported by ≥10 reads were plotted as a circular diagram illustrated by Figure 3 for BMV and Figure 4 for CCMV. The connecting lines represent recombination events within and between different RNA segments, whereas the concentric outer ring histograms summarize the frequency of intra- and inter-recombination events, multi-base substitutions, and SNPs, respectively. CCMV reveals more hotspots than BMV, particularly in RNA3 at several locations. Examples in Figure 3, Figure 4 and Figure S2 illustrate the distribution of hotspots on BMV-RNA1 (position 1097) versus CCMV-RNA1 (1103) (Table 6). The hotspots for intra- and inter-crossovers were scattered in CCMV RNA1 at positions 1231, 1260 and 1261; CCMV-RNA2 at 2016 has hot spots for both inter- and intra-crosses (Table 6). Interestingly, in CCMV, some of the highest hotspot peaks are also found on the multi-base substitution ring (purple ring in Figure 4), suggesting that these regions are highly mutable. Further studies are required to explain the observed differences between BMV and CCMV.

### 3.6. Recombination Events between Virus and Host RNAs

Table 2 and Table 3 show that a small percentage of BMV and CCMV recombinant reads (respectively 0.07% and 0.04%) mapped to their respective host mRNAs or rRNAs. In addition, there were host-derived reads carrying mutations parsed from the ViReMa output files. There were 170 (BMV) and 138 (CCMV) host–host or virus–host cross events (see detailed breakdown of numbers in Table 7 (count of events) and Table S2 (count of reads)), Generally, BMV carried more virus–host than host–host recombinants than did CCMV. Interestingly, micro-insertions were not found in the host sequences. Surprisingly, 2.21% host–host cross events in CCMV (as shown in Table 7) were covered by 28.31% of reads (Table S2), suggesting some enhancing processes or possible PCR chimera artifacts. To resolve the two possibilities, additional work will be needed in the future to include control experiments with mixed-host RNAs and encapsidated viral RNAs for sequencing without the PCR amplification. Also, among the BMV virus-mRNA cross events, 76 occurred at the intercistronic region (positions 1185–1200) (NC_002028.1 pink peak, Figure 3). In addition to recombinants, there was a higher percentage (0.1%) reads mapped just to host RNAs, extending previous results about the ability of packaging of host RNAs in virus particles (6).

## 4. Discussion

In this work, we characterize a population of the encapsidated RNAs in two bromoviruses, BMV and CCMV. Among several software packages available, we focused on the program ViReMa that initially finds a seed-based alignment, and then identifies a new read segment at the 3′ side vis-á-vis the reference genomes. This then detects multiple recombinants within a read, including insertions and substitutions at the junctions [26]. Various types of RNA recombinants were detected, from unambiguous recombinants to the unidentifiable (ambiguous) variants. For both viruses, a vast majority of reads was mapped to the viral genomes but some to the host cellular RNAs, as shown in Table 2.

Our previous studies on homologous recombination using two BMV strains [17] revealed recombination hot spots between marker mutations mostly at the coding regions in RNAs 1 and 2. In RNA3, the crosses occurred within both the inter-cistronic region and the 3a open reading frame (ORF) (the CP-binding region) but were much lower within the CP ORF. Since the marker mutations designed were positioned far apart, mapping of the crossover sites to a narrow region was not possible. Here, we narrowed the cross sites by using HTS of the encapsidated RNAs. The major clusters of crosses were mapped at multiple spots (Table 6) within the ORFs but less within the untranslated regions (UTRs.) Lower number of crosses at the UTRs were at the conserved sequences, which is likely associated with the functional relevance of the regions (see below).

Viral and host factors can impact recombination activity at hot spots [32,33,34,35,36]. We demonstrated previously that binding of CP to cis-acting RNA motifs debilitated recombination in BMV RNA3 [22]. Also, the structure of viral RNAs per se can affect cross sites, as shown for HIV-1 [37] or BMV [38]. Yet selection pressure for functional sequences is also important. For instance, portions of the 3′ UTR and around the middle of 3a ORF in BMV RNA3 serve as the encapsidation and RNA replication signals [39], and indeed they support lower crossover frequency, shown in Figure 3. Also, reduced crosses occurred at the mapped RNA3 replication enhancer site [40], positions 1043–1167. Other modifications such as SNPs, insertions/substitutions, or inter-segmental crosses as shown in Figure 3 and Figure 4 are also reduced within these regions, which suggests the functionality of the preserved sequences.

Two major categories of recombinants were the intra-segmental and the inter-segmental variants. The majority of BMV recombinants, as shown in Table 4 and Table S1, were intra-segmental. Circos plots represented by Figure 3 and Figure 4 illustrate the crosses in a global form. Intersegmental crosses were less frequent but detectable, with the most apparent ones within the RNA1 and RNA2 ORFs (Table 4), but less frequent between the 3′ UTRs of RNA2 and RNA3 (Figure 3). The latter might reflect a negative selection for recombinants during encapsidation because the 3′ UTRs contain both the encapsidation (at least for BMV RNA3) and the replication initiation signals. Also, the BMV RNA3 ORFs were less active than for CCMV, likely reflecting some differences in requirements for host factors between two viruses [41]. In addition, in this work we observed disparities between BMV and CCMV regarding viral–host recombinants (Table 7, Table S2, and Figure S2), perhaps due to different host contributions. Interestingly, the crosses between virus and host rRNAs were very limited despite known abundant concentration of rRNAs (Table 7 and Table S2), possibly reflecting the separation of recombination and translation functions. Overall, there are limited reports about viral–host RNA recombination events [4,42,43].

Reads that carried micro-insertions (less than 4 nts long) were identified at about 50 times higher in CCMV compared to BMV, as shown in Table 3. However, the number of events encompassed within these reads were similar for the two viruses (28 insertions in BMV and 33 in CCMV: Table 7). These non-templated insertions might imply that either double (multiple) crosses with host RNAs or some form of reiterative RNA synthesis occurred more in CCMV, possibly because of less precise CCMV replicase and/or RNA editing in comparison to BMV. Apparently, CCMV seems more prone to supporting insertions in its genomic RNAs. In contrast to insertions, for both viruses, there were similar numbers of reads carrying two or more nucleotide substitutions as shown in Table 3 and Table 7. The mechanism of such replacements is probably different from that for micro-insertions and might involve a precise process, e.g., RNA re-ligation with non-templated copying.

Single nucleotide substitutions (SNPs) were mapped in all of the RNA segments of both viruses (Figure 3 and Figure 4), with the highest number for BMV RNA3 (in CP ORF) and for CCMV RNA2 (in 2a ORF).

A number of reads (4.21% of total reads in BMV and 5.20% in CCMV) remained unmapped to the viral genome and to host references (Table 3), and hence their origin is unclear. Possibilities include (i) reads from parts of the host’s genome other than the transcriptome (we used only mRNAs and tRNAs); (ii) low-quality reads; or (iii) contaminated reads.

In summary, these studies reveal the dynamic character of the viral RNA genome, generating the encapsidated RNA variants. A question remains whether the HTS methodology detects false-positive reads, as described for other cases [28,44]. In this work, we attempted to reduce potential noise by requiring that a junction be represented by at least ten overlapping reads (Table 4 and Table 7). Future analyses with other controls must be used to eliminate more false positives. One baseline control would involve mixing separate RNA samples preceding the reverse transcription reaction. Nevertheless, our findings encourage the use of these novel approaches by inspiring a closer look at where the viral RNAs recombine and what types of events predominate. In addition, this methodology can be used to reveal potentially functional domains in viral RNAs, helpful for novel anti-viral therapies via targeting recombination hotspots or functional motifs.

## Figures and Tables

**Table 1:** Reference genome statistics.

GenBank ID	RNA Segment	Length (nts)	Encoded Proteins and nts Positions
**BMV**			
NC_002026.1	RNA1	3234	1a (transferase-helicase): 75–960
NC_002027.1	RNA2	2865	2a (RdRp): 104–2572
NC_002028.2	RNA3	2111	3a (movement): 92-1003; coat: 1245–1814
Host: *H. vulgare* ^1^	248,180 mRNAs	Total 478,475,198	
	1347 rRNAs	Total 1,491,619	
**CCMV**			
AF325739.1	RNA1	3174	1a: 71–2950
AF325740.1	RNA2	2773	2a: 109–2535
AF325741.1	RNA3	2175	3a: 239–1147, coat: 1362–1934
Host: *V. unguiculata* ^2^	42,287 mRNAs	Total 83,574,877	
	22 rRNAs	Total 12,051	

^1^ Phytozome genome ID: 462, ^2^ Phytozome genome ID: 469.

**Table 2:** Bowtie mapping results using all reads as input.

Read Mapping ^a^	BMV	CCMV
Total no. reads	108,930,036	108,937,068
Mapped to RNA1 (%)	24,647,631 (22.63%)	37,785,834 (34.69%)
Mapped to RNA2 (%)	43,755,190 (40.17%)	17,519,764 (16.08%)
Mapped to RNA3 (%)	9,022,371 (8.28%)	18,284,856 (16.78%)
Mapped to host mRNAs (%)	75,289 (0.07%)	43,928 (0.04%)
Mapped to host rRNAs (%)	348 (0%)	110,387 (0.1%)
Unmapped reads (%)	31,429,207 (28.85%)	35,192,299 (32.31%)

^a^ The total reads were mapped to respective references with no mismatches allowed.

**Table 3:** Bowtie-unmapped reads analyzed and classified by ViReMa.

ViReMa Analyzed Reads ^a^	BMV + (Host mRNAs and rRNAs)	CCMV + (Host mRNAs and rRNAs)
Total reads analyzed	31,429,207	35,192,299
Recombinations	440,975	644,596
	1.40%	1.83%
Nucleotide substitutions (≥2 nt)	391,820	426,030
	1.25%	1.21%
Micro-insertions (≤4 nt)	1616	81,264
	0.01%	0.23%
Single mapping reads with pads	25,268,730	28,884,471
	80.40%	82.08%
Ambiguous recombinations	4,000,122	3,326,635
	12.73%	9.45%
Completely unmapped	1,322,860	1,828,745
	4.21%	5.20%

^a^ The inputs to ViReMa were the unmapped reads from the Bowtie step.

**Table 4:** Counts of recombination events among different RNA segments of BMV and CCMV.

BMV				
Column is 5′ & Row is 3′	RNA1	RNA2	RNA3	Total
RNA1	1183	78	22	1283
RNA2	176 ^a^	2046	28	2250
RNA3	37	24	415	476
Total	1396	2148	465	4009
**CCMV**				
Column is 5′ & row is 3′	RNA1	RNA2	RNA3	Total
RNA1	1726	175	234	2135
RNA2	165	949	141	1255
RNA3	247	133	1213	1593
Total	2138	1257	1588	4983

The table represents the number of events supported by ≥10 reads. ^a^ For example, 176 represents the number of recombination reads between RNA1 and RNA2. Each of the 176 events has a different starting position in RNA1 or end position in RNA2. Note here, an event differs from a read: one event refers to one recombination junction in the reference, specified by a starting position in one RNA molecule and an ending position in another RNA molecule, which can be supported by multiple reads. For events supported with ≥5 reads, see Table S1.

**Table 5:** Indel types and frame shifts caused by intra-recombination events.

Indel Types ^a^	Frame Shifts ^b^	BMV		CCMV	
		Counts	%	Counts	%
del3	1->1	178	4.89	207	5.32
ins3	1->1	506	13.89	379	9.75
del1	1->2	1560	42.83	1775	45.65
ins2	1->2	433	11.89	405	10.42
del2	1->3	411	11.29	541	13.91
ins1	1->3	554	15.21	581	14.94
Total events		3642		3888	

^a^ Del3 means the number of deleted bases is the multiple of 3, del1 means the number of deleted bases is the multiple of 3 plus 1, and del2 means the number of deleted bases is the multiple of 3 plus 2. ^b^ 1->1 means if the indel occurs inside the protein coding region, the reading frame will not change (from frame 1 to frame 1); 1->2 and 1->3 means the reading frame will change frame 1 respectively to frame 2 or frame 3.

**Table 6:** Recombination hotspots in BMV and CCMV.

BMV	Intra-Recombination Hotspot Position (Number of Events ^a^)	Inter-Recombination Hotspot Position (Number of Events)
RNA1	1054 (13), 1058 (13), 1097 (14), 1360 (14)	1053 (11), 1054 (22),1058 (15), 1097 (15)
RNA2	1212 (17), 1274 (11), 1314 (15), 1488 (20), 1559 (24), 1773 (36), 1776 (21), 1792 (15), 1795 (16)	na
RNA3	na	1200 (23)
**CCMV**	**Intra Recombination Hotspot Position (Number of Events)**	**Inter Recombination Hotspot Position (Number of Events)**
RNA1	815 (21), 877 (13), 888 (12), 1104 (15), 1231 (35), 1103 (12), 60 (22), 1261 (12)	734 (11), 1104 (14), 1229 (18), 1230 (13), 1231 (82), 1260 (38), 1261 (30)
RNA2	653 (16), 2016 (40), 2017 (20), 2018 (12)	852 (11), 2016 (43), 2017 (15)
RNA	359 (15), 906 (11), 950 (26), 1585 (27), 1586 (11), 1731 (15), 1801 (14)	359 (15), 1584 (25), 1585 (29), 1586 (16), 1801 (18)

^a^ Only events supported by ≥10 reads were counted.

**Table 7:** Breakdown counts of mutation events supported by ≥10 reads*.

Recombinations	BMV	BMV (%)	CCMV	CCMV (%)
Virus–Virus	4009	95.93	4983	97.31
mRNA–mRNA	91	2.18	113	2.21
rRNA–rRNA	0	0.00	12	0.23
Virus–mRNA	76	1.82	8	0.16
Virus–rRNA	3	0.07	4	0.08
mRNA–rRNA	0	0.00	1	0.02
Substitutions (≥2 nt)	BMV	BMV (%)	CCMV	CCMV (%)
Virus–Virus	4679	99.98	4114	97.77
mRNA–mRNA	0	0	0	0
rRNA–rRNA	1	0.02	94	2.23
Micro–insertions (≤4 nt)	BMV	BMV (%)	CCMV	CCMV (%)
Virus–Virus	28	100	33	100%
mRNA–mRNA	0	0	0	0%
rRNA–rRNA	0	0	0	0%
Ambiguous recombinations	BMV	BMV (%)	CCMV	CCMV (%)
Virus–Virus	33,924	86.33	33,960	94.27
mRNA–mRNA	4829	12.29	1342	3.73
rRNA–rRNA	104	0.26	679	1.88
Virus–mRNA	243	0.62	44	0.12
Virus–rRNA	192	0.49	0	0
mRNA–rRNA	4	0.01	0	0
Single mapping	BMV	BMV (%)	CCMV	CCMV (%)
Virus–Virus	194,281	99.62	229,656	99.43
mRNA–mRNA	732	0.38	250	0.11
rRNA–rRNA	0	0	1056	0.46

## Data Availability

The cleaned BMV and CCMV reads were deposited into GenBank (PRJNA565451): https://www.ncbi.nlm.nih.gov/sra/SRX6849035 for CCMV and https://www.ncbi.nlm.nih.gov/sra/SRX6849034 for BMV.

## Supplementary Materials

The following supporting information can be downloaded at: https://www.mdpi.com/article/10.3390/pathogens13010096/s1, Figure S1: Length distribution of indels caused by intra-segmental recombination in BMV and CCMV.; Figure S2: An example of recombination hotspots in BMV and CCMV RNA1. Table S1: Counts of recombination events among different RNA segments of BMV and CCMV; Table S2: Breakdown counts of reads of mutation events supported by ≥10 reads.
