# Supplementary material for: Characterization of Variant RNAs Encapsidated during Bromovirus Infection by High-Throughput Sequencing
Source: Pathogens. 2024 Jan 22;13(1):96. doi: 10.3390/pathogens13010096 (PMC10819421; doi:10.3390/pathogens13010096)
Supplement: Supplementary file 1 [file pathogens-13-00096-s001.zip › pathogens-2732810-supplementary.pdf]

pathogens

MDPI

Supplementary Materials

Table S1. Counts of recombination events among different RNA segments of BMV and CCMV.

		BMV		
**Column is 5′ & row is 3′**	**RNA1**	**RNA2**	**RNA3**	Total
RNA1	**2834**	407	67	3308
RNA2	751	**4511**	118	5380
RNA3	138	132	**908**	1178
Total	3723	5050	1093	**9866**
		**CCMV**		
**Column is 5′ & row is 3′**	**RNA1**	**RNA2**	**RNA3**	**Total**
RNA1	**3390**	496	653	4539
RNA2	442	**1898**	365	2705
RNA3	655	365	**2501**	3521
Total	4487	2759	3519	**10765**

[a] These numbers are the counts of events supported by ≥5 supporting reads. For example, 300 are the number of recombination events supported by reads with their 5′ end mapped to RNA1(+ strand) and 3′ end mapped to RNA2 (+ strand). Each of the 300 events has a different starting position in RNA1(+) or end position in RNA2(+).

Table S2. Breakdown counts of reads of mutation events supported by ≥ 10 reads.

**Recombinations**	**BMV**	**BMV (%)**	**CCMV**	**CCMV (%)**
Virus–Virus	331,572	98.78%	291,721	71.03%
mRNA–mRNA	1346	0.40%	116,273	28.31%
rRNA–rRNA	0	0.00%	1917	0.47%
Virus–mRNA	2718	0.81%	169	0.04%
Virus–rRNA	43	0.01%	161	0.04%
mRNA–rRNA	0	0.00%	435	0.11%
Substitutions (≥2 nt)	BMV	BMV (%)	CCMV	CCMV (%)
Virus–Virus	132,863	99.99 %	136,258	74.74 %
mRNA–mRNA	0	0 %	0	0 %
rRNA–rRNA	12	0.01 %	46,041	25.26 %
Micro-insertions (≤4 nt)	BMV	BMV (%)	CCMV	CCMV (%)
Virus–Virus	1050	100 %	80,647	100 %
mRNA–mRNA	0	0 %	0	0 %
rRNA–rRNA	0	0 %	0	0 %
Ambiguous recombinations	BMV	BMV (%)	CCMV	CCMV (%)
Virus–Virus	3,041,044	88.76 %	2,315,504	86.4 %
mRNA–mRNA	241,072	7.04 %	173,000	6.45 %
rRNA–rRNA	119,073	3.48 %	190,549	7.11 %
Virus–mRNA	4,527	0.13 %	1,080	0.04 %
Virus–rRNA	19,772	0.58 %	0	0 %
mRNA - rRNA	701	0.02 %	0	0 %
Single mapping	BMV	BMV (%)	CCMV	CCMV (%)
Virus–Virus	25,127,157	99.94 %	28,588,383	99.41 %
mRNA–mRNA	15,802	0.06 %	21,719	0.08 %
rRNA–rRNA	0	0 %	147,055	0.51 %

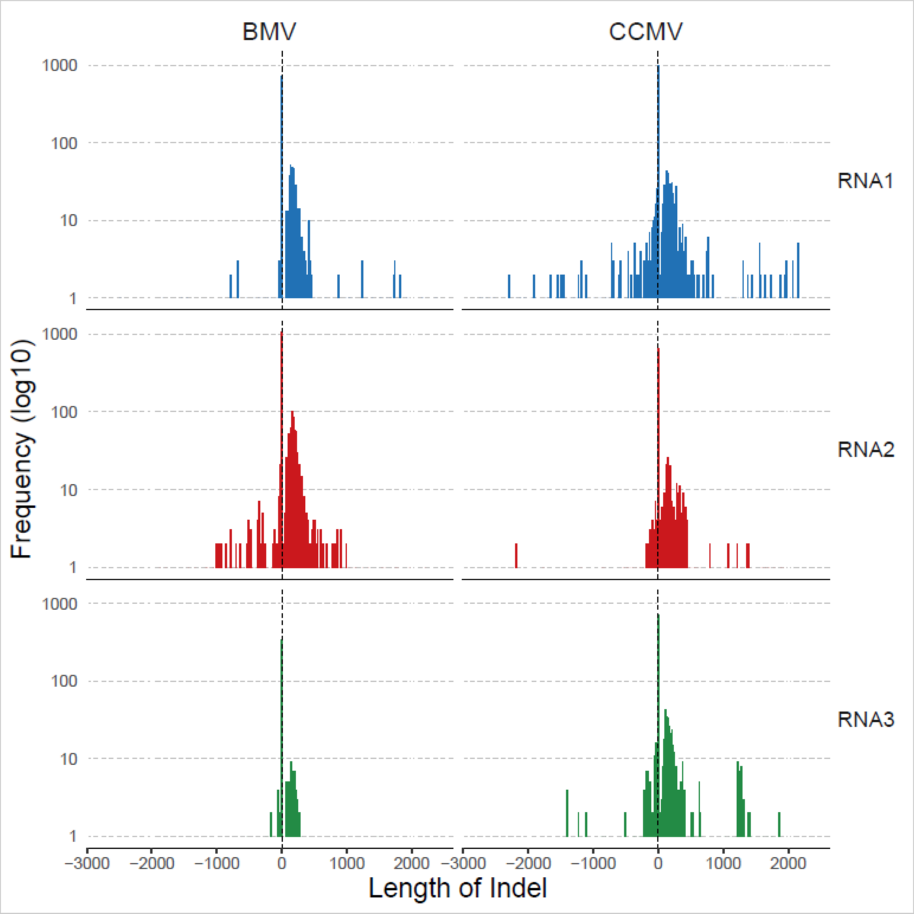

Figure S1. Length distribution of indels caused by intra-segmental recombination in BMV and CCMV. The x-axis is length of indels (negative values are deletions and positive values are insertions) and y-axis is the log10 of the frequency. In both viruses, there is a highest peak at x = −1 (very close to the dashed line x = 0), meaning deletion size = 1. For this peak, the frequency in y-axis is equal to 1477 and 1639, respectively, in BMV and CCMV. Therefore, there are actually more deletions than insertions in the plot.

```
BMV                 1097
          -----|---------|---------|---------|---------|---------|
     1085 agaggtagaggagatagctttcagatgtttcaaggaaagtaaagaatggactgaga 1140
      338  E  V  E  E  I  A  F  R  C  F  K  E  S  K  E  W  T  E  N 356

CCMV                          1103
          ---------|---------|---------|---------|---------|------
     1081 agaggttgaagagattgcttttcgatgttttaaggagaataaagagtggacggaga 1136
      338  E  V  E  E  I  A  F  R  C  F  K  E  N  K  E  W  T  E  N 356
```

Figure S2. An example of recombination hotspots in BMV and CCMV RNA1. The numbers are the nucleotide positions in the RNA1 (+) of the two viruses. The rulers are shown on the top, the nucleotide sequence in the middle (numbers are nt positions) and the amino acid translation in the bottom (numbers are aa positions). The two nucleotide regions are shown as if they are pair-wise aligned, with the two hotspot positions and nucleotide bases highlighted in red.
